# *Bifidobacterium* and *Lactobacillus* Counts in the Gut Microbiota of Patients With Bipolar Disorder and Healthy Controls

**DOI:** 10.3389/fpsyt.2018.00730

**Published:** 2019-01-18

**Authors:** Emiko Aizawa, Hirokazu Tsuji, Takashi Asahara, Takuya Takahashi, Toshiya Teraishi, Sumiko Yoshida, Norie Koga, Kotaro Hattori, Miho Ota, Hiroshi Kunugi

**Affiliations:** ^1^Department of Mental Disorder Research, National Institute of Neuroscience, National Center of Neurology and Psychiatry, Tokyo, Japan; ^2^Department of Human Life Science, Nagoya University of Economics, Aichi, Japan; ^3^Yakult Central Institute, Tokyo, Japan; ^4^Department of Psychiatry, National Center of Neurology and Psychiatry Hospital, Tokyo, Japan

**Keywords:** *Bifidobacterium*, *Lactobacillus*, bipolar disorder, cortisol levels, stress response

## Abstract

**Background:** Although the pathophysiology of bipolar disorder remains elusive, growing evidence suggests the beneficial effects of *Bifidobacterium* and *Lactobacillus* in the gut microbiota on stress response and depressive symptoms. In the present study, we examined *Bifidobacterium* and *Lactobacillus* counts for association with bipolar disorder and serum cortisol levels.

**Methods:** Bacterial counts in fecal samples were examined in 39 patients with bipolar disorder according to the Diagnostic and Statistical Manual of Mental Disorders, 4th edn. and 58 healthy controls using bacterial rRNA-targeted reverse transcription-quantitative polymerase chain reaction.

**Results:** No significant difference was found in either bacterial counts between the two groups. However, we found a significantly negative correlation between *Lactobacillus* counts and sleep (ρ = −0.45, *P* = 0.01). Furthermore, a significant negative correlation was found between *Bifidobacterium* counts and cortisol levels (ρ = −0.39, *P* = 0.02) in the patients, although such a correlation was not found for *Lactobacillus* counts.

**Conclusions:** Our results suggest that *Bifidobacterium* or *Lactobacillus* counts may not play a major role in the pathophysiology of bipolar disorder in our sample. However, the observed negative correlation between *Lactobacillus* counts and sleep and that between *Bifidobacterium* counts and serum cortisol levels point to the possible roles of these bacteria in sleep and stress response of the patients.

## Introduction

Increasing evidence suggests that alterations in the gut microbiota are involved in stress-related psychiatric disorders (1–4). Animal studies have revealed that the enteric microbiota affects neural activity, neuronal pathways, emotional state, immune system/inflammation, stress responses (i.e., hypothalamic-pituitary-adrenal [HPA] axis activity), and cognitive function in the host. A recent study reported that fecal microbiota transplantation from depressed patients causes depression-like behaviors in mice (5–11).

Bipolar disorder is a serious mental disorder that markedly affects an individual's daily life. The efficacy of current pharmacological treatment is inadequate; in brief, residual mood symptoms often remain after initial treatment and subsequent recurrence is frequent, which might be due to the lack of knowledge regarding the neurobiological mechanisms underlying the disorder (12, 13). Nonetheless, bipolar disorder is considered to be caused by abnormal monoamine functions, dysregulation of the hypothalamus-pituitary-adrenal (HPA) axis, and chronic inflammation (14–17), all of which may be mediated by alterations in the gut microbiota (8, 9, 11, 18).

Amongst the many bacterial genera in the gut, *Bifidobacterium* and *Lactobacillus* are considered to be probiotics, which exert beneficial effects on physical and mental health such as psychiatric symptoms. For example, Messaoudi et al. (19) reported that healthy volunteers, who consumed a combination of *Bifidobacterium longum* R0175 and *Lactobacillus helveticus* R0052, exhibited less distress symptoms and showed a decrease in urinary free cortisol levels (19), suggesting ameliorating effects of the probiotics on the stress-related hormonal system. The same probiotics also had a beneficial effect on brain plasticity in mice exposed to chronic stress (7). Desbonnet et al. (20) reported that *Bifidobacterium infantis* treatment normalized the immune response and ameliorated depression-like behavior in a rat maternal separation model of depression. Although direct evidence in psychiatric patients is still scarce, we have recently reported that *Bifidobacterium* and *Lactobacillus* counts tend to be lower in patients with major depressive disorder than in healthy controls, supporting the possible role of these bacteria in the pathophysiology of mood disorders (21). Furthermore, a randomized clinical trial showed that taking probiotic capsules (*L. acidophilus, L. casei*, and *Bifidobacterium bifidum*) had a beneficial effect on depressive symptoms, glucose metabolism, and antioxidant action (1).

Recently, several research groups have reported gut microbiota in patients with bipolar disorder (22). Evans et al. (23) examined 115 patients with bipolar disorder and 64 controls, recruited from the University of Michigan, using 16S ribosomal RNA (rRNA) gene sequence analysis and Operational Taxonomical Unit (OTU) level analysis. They found significantly decreased fractional representation of *Faecalibacterium* in bipolar disorder patients (23). The same research group compared the gut microbiota between 46 atypical antipsychotic-treated and 69 untreated patients and found differences in OTU levels (24). Painold et al. (25) performed 16S rRNA gene sequencing of stool samples from 32 bipolar individuals and 10 controls recruited from the Medical University of Graz, Austria. They reported that the phylum *Actinobacteria* and the class *Coriobacteria* were significantly more abundant in bipolar patients than in controls, while *Ruminococcaceae* and *Faecalibacterium* were more abundant in healthy controls. That the results of the previous two study groups were inconsistent, warrants further investigation. Additionally, given that similar studies have not been conducted with Asian bipolar patients, warrants further research in Asian populations, particularly because dietary differences have a substantial impact on gut microbiota (25). In the current study, we examined whether *Bifidobacterium* and *Lactobacillus* counts are altered in Japanese patients with bipolar disorder in comparison with healthy controls. We focused on these two bacteria because research has suggested that they have a beneficial effect on pathological factors related to mood disorders (1, 7, 19, 20, 26). In addition, we examined the possible correlation between the bacterial counts and clinical variables such as severity of symptoms, medication, and serum cortisol levels.

## Materials and Methods

### Participants

We recruited 39 patients with bipolar disorder (13 bipolar I and 26 bipolar II) and 58 healthy controls from the outpatient clinic at the National Center of Neurology and Psychiatry (NCNP) hospital and from the local community (Western Tokyo) through the NCNP website and local magazine advertisements. The majority of the controls had participated in our previous study (21). All participants were biologically unrelated Japanese individuals who were screened for eligibility by a research psychiatrist using the Japanese version of the Mini-International Neuropsychiatric Interview (27, 28), and an additional unstructured interview, to obtain information on education, illness history, psychiatric medication, height and weight, comorbid medical conditions, and recent use of antibiotics. The diagnosis of bipolar disorder was made by a board-certificated research psychiatrist, according to the Diagnostic and Statistical Manual of Mental Disorders, 4th ed. (DSM-IV) (29), based on information obtained from the interviews and medical records, if available. Depressive symptoms of patients with bipolar disorder were rated using the 17-item version Hamilton Depression Rating Scale (HAM-D) (30), while the Young Mania Rating Scale was used to assess manic symptoms (31). The control group was screened to exclude candidates with a current or past history of psychiatric illness or contact with psychiatric services. Exclusion criteria for both patients and control groups included a prior medical history of central nervous system disease, including epilepsy and severe head injury, substance abuse or dependence, mental retardation, recent use of antibiotics, history of gastrointestinal surgery, severe congenital abnormalities, or any severe medical conditions. Among the patients with bipolar disorder (*n* = 36), 33 patients were receiving pharmacological treatment, while the remaining three were not. The doses of antidepressants and antipsychotics were converted to imipramine and chlorpromazine equivalents, respectively, using the published guideline (32). Moreover, we obtained information on the patients' use of mood stabilizing medicine, such as lithium, valproate, lamotrigine, and carbamazepine. We obtained information on intake of probiotic drugs (prescribed or obtained over the counter); 9 patients were on probiotic medication. The present study was approved by the ethics committee of the National Center of Neurology and Psychiatry. Written informed consent was obtained from all participants prior to their enrollment in the study.

### Collection of Fecal Samples

Participants were asked to place a fecal sample of approximately 1 g into a sample tube containing 2 mL of RNA stabilization solution, Ambion RNA*later*® (Thermo Fisher Scientific, Waltham, MA, United States). The collected samples were kept at 4°C in a refrigerator. All fecal samples were subsequently sent to the Yakult Central Institute and stored at 4°C until assay. Samples were weighed, and RNA*later*® was added to the tube in order to make a 50-fold diluted (v/w) fecal homogenate by vortex. This fecal homogenate (200 μl) was then transferred into a 2-ml screw-cap microtube and centrifuged at 12,000 × g for 5 min at 4°C, and the supernatant was discarded by decantation. The pellet was then stored at −80°C until extraction of RNA.

### Determination of Bacterial Counts

Bacterial counts in fecal samples were measured by investigators blinded to the clinical information. The measurements were performed using the Yakult Intestinal Flora-SCAN, which is based on a 16S or 23S rRNA-targeted reverse transcription-quantitative polymerase chain reaction (RT-qPCR), to determine the composition of major gut bacterial groups according to a previously described method (33, 34). Briefly, total RNA fractions were extracted from the fecal samples, and three serial dilutions of the extracted RNA sample were used for bacterial rRNA-targeted RT-qPCR. The threshold cycle values in the linear range of the assay were applied to the standard curve to obtain the corresponding bacterial count in each nucleic acid sample. Those data were subsequently used to determine the number of bacteria per sample. The specificity of the RT-qPCR assay, using group-, genus-, or species-specific primers, was determined as previously described (33, 34). *Bifidobacterium* counts were determined using a single primer set. *Lactobacillus* counts were determined by adding organisms of six *Lactobacillus* subgroups (*L. casei, L. gasseri, L. plantarum, L. reuteri, L. ruminis*, and *L. sakei*) and two *Lactobacillus* species (*L. brevis* and *L. fermentum*) together, which were determined using separate RT-qPCR primers. Briefly, for identification of the target bacterial population in the fecal samples, 1/20,000, 1/200,000, or 1/2,000,000 samples of the extracted RNA from 4 mg of wet feces was subjected to RT-qPCR. The quantification cycle values in the linear range of the assay were applied to the analytical curve, generated in the same experiment, to obtain the corresponding bacterial count using the 4′,6-diamidino-2-phenylindole (DAPI) staining method in each nucleic acid sample. Bacterial counts were then converted to count per sample.

### Blood Sample Collection

Fasting venous blood samples were collected between 9:00 and 10:00 a.m. from each participant. Samples were collected in a 9-ml serum separating tube and centrifuged at 3,000 rpm for 10 min. Cortisol levels were measured via radioimmunoassay at SRL Inc. (Tokyo, Japan).

### Statistical Analyses

Data are presented as means ± standard deviation (*SD*) unless otherwise specified. Demographic and clinical variables were compared using the chi-square test for categorical variables and the *t*-test for continuous variables. Univariate analysis of covariance (ANCOVA) was used to compare *Bifidobacterium* or *Lactobacillus* counts between patients with bipolar disorder and controls (adjusted for age and sex). Additional analyses controlling for BMI were also performed. Moreover, we performed ANCOVA, controlling for age, sex, and diagnosis in order to examine the influence of bacterial counts on levels of cortisol. Partial correlation analysis (adjusted for age and sex) was used to examine the partial correlations between bacterial counts and other variables such as HAM-D scores and cortisol levels. Cramer's *V* was calculated as a measure of effect size for χ^2^ tests, *d* for *t*-tests, η^2^ for ANOVA, and partial η^2^ for ANCOVA. Differences were considered statistically significant when the two-tailed *P* value was < 0.05. Analysis was performed using the Statistical Package for the Social Sciences version 21.0 (IBM Corp, Chicago, Illinois, USA).

## Results

### Characteristics of Study Participants

Table 1 lists the demographic and clinical characteristics of the participants. No significant differences were observed in age, sex, body mass index (BMI), or years of education between the patient and control groups. The number of patients with depressed, manic, euthymic, or mixed episode according to the standard cut-off scores (HAM-D17 score of 7 and YMRS score of 7) were 23, 2, 13, and 1, respectively. Thus, the majority of the subjects were depressed or euthymic and there were only three patients whose YMRS score was eight or more.

**Table 1:** Demographic and clinical characteristics of the participants.

	**Bipolar disorder (*n* = 39)**	**Control (*n* = 58)**	**Bipolar disorder vs. control**
Male (%)	17 (44)	22 (38)	χ^2^ = 0.31, *df* = 1, *P* = 0.58, Cramer's *V* = 0.06
Age, years:	40.3 ± 9.2	43.1 ± 12.9	t = 1.23, *df* = 94.6, *P* = 0.22, *d* = 0.24
Education, years	15.3 ± 3.3	15.2 ± 2.7	t = −0.14, *df* = 95, *P* = 0.89, *d* = 0.03
Body mass index (BMI)	23.9 ± 4.7	22.4 ± 3.8	t = −1.71, df = 69.9, *P* = 0.09, *d* = 0.36
HAM-D 17 total score	10.3 ± 7.0	N.A.
Young Mania Rating Scale	2.1 ± 3.5	N.A.
Age at onset, years	28.2 ± 9.4	N.A.
Medication *n* (%) mg/day		
Antipsychotic^#^*n* = 13 (33%)	182.9 ± 179.9	N.A
Antidepressant^##^*n* = 12 (31%)	204.3 ± 125.8	N.A
Lithium *n* = 16 (41%)	418.8 ± 240.1	N.A
Sodium Valproate *n* = 8 (21%)	725.0 ± 399.1	N.A
Lamotrigine *n* = 13 (33%)	186.5 ± 123.2	N.A
Carbamazepine *n* = 4 (10%)	325.0 ± 221.7	N.A

*Values are mean ± standard deviation*.

*N.A, not applicable; HAM-D, Hamilton Depression Rating Scale*.

#: *Chlorpromazine equivalent dose in patients with antipsychotic medication (Bipolar disorder, n = 13)*.

## *: Imipramine equivalent dose in patients with antidepressant medication (Bipolar disorder, n = 12)*.

*Drug free, n = 3*.

### Comparison of Bacterial Counts

**Figure 1:**
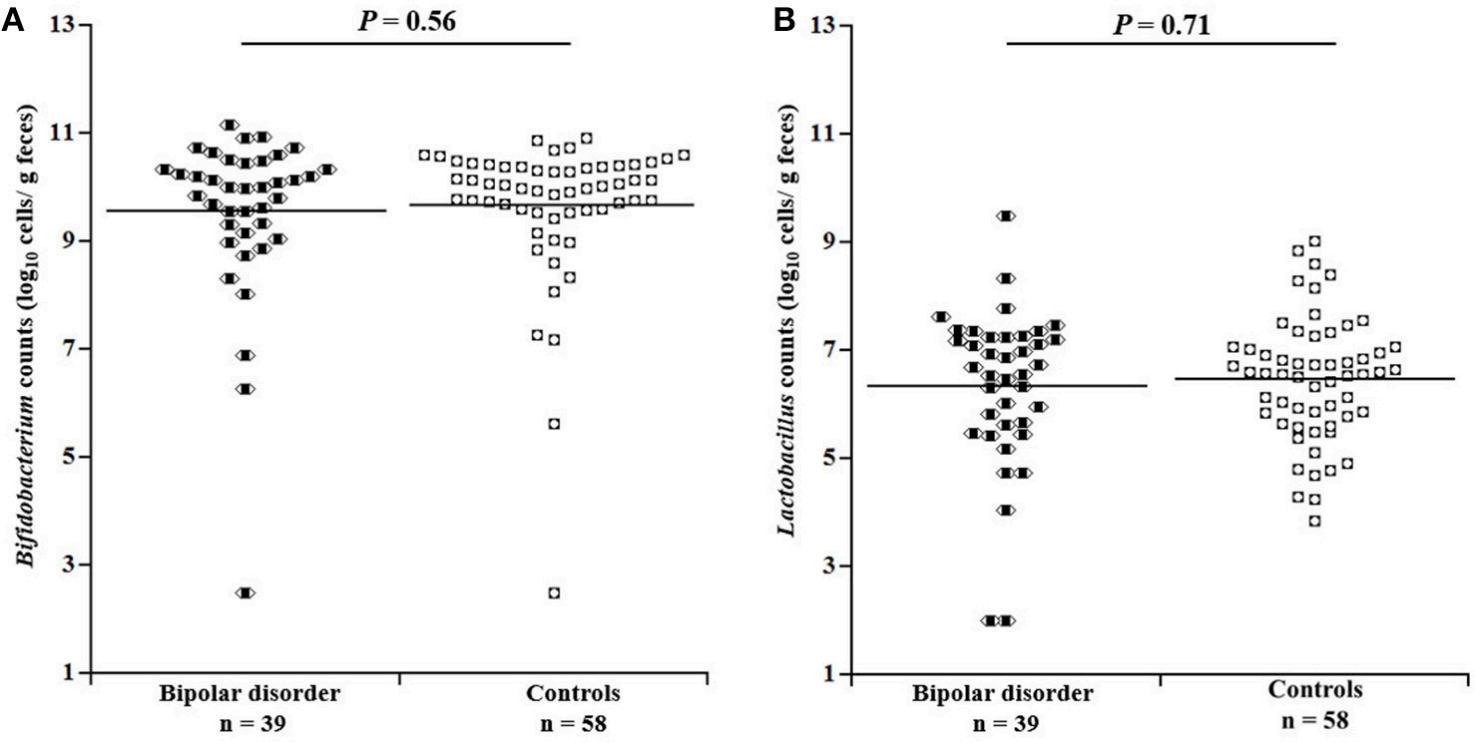
Comparison of bacterial counts in the gut microbiota between patients with bipolar disorder and controls. No significant differences were observed between patients and controls with respect to either **(A)** *Bifidobacterium* (*df* = 1, 92; *F* = 0.34, *P* = 0.56, Partial ρ^2^ = 0.004, ANCOVA) or **(B)** *Lactobacillus* (*df* = 1, 92; *F* = 0.14, *P* = 0.71, Partial ρ^2^ = 0.002) counts.

Figure 1 depicts comparisons of *Bifidobacterium* and *Lactobacillus* counts between the patients and controls. No significant differences were found in *Bifidobacterium* (*df* = 1, 92; *F* = 0.34, *P* = 0.56, Partial η^2^ = 0.004) or *Lactobacillus* counts (*df* = 1, 92; *F* = 0.14, *P* = 0.71, Partial η^2^ = 0.002) between the two groups. Even when male and female were examined separately, there was no significant difference in bacterial counts between the patients and controls (Supplementary Figure 1). When bipolar I and II patients were separately compared with controls, there was no significant difference in *Bifidobacterium* counts for bipolar I (*df* = 1, 66; *F* = 0.05, *P* = 0.83, Partial η^2^ = 0.001) or for bipolar II (*df* = 1,79; *F* = 0.83, *P* = 0.36, Partial η^2^ = 0.01) or in *Lactobacillus* counts for bipolar I (*df* = 1, 66; F = 0.46, *P* = 0.50, Partial η^2^ = 0.01) or for bipolar II (*df* = 1, 79; *F* = 0.86, *P* = 0.36, Partial η^2^ = 0.01). When BMI was additionally controlled for, the results were essentially unchanged (data not shown).

### Bacterial Counts and Symptom Scores

**Figure 2:**
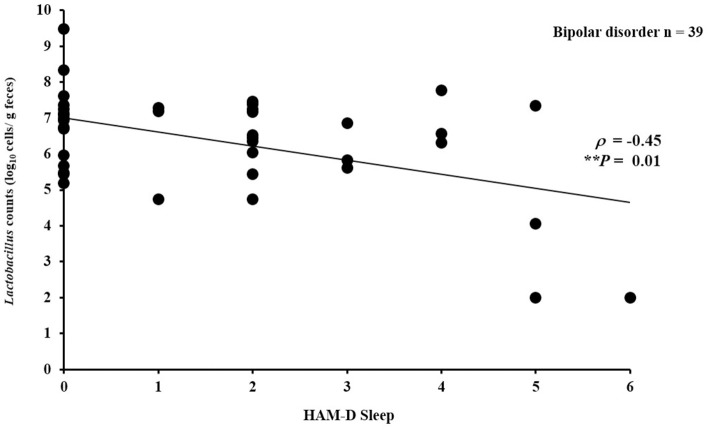
Correlation between *Lactobacillus* counts and sleep (HAM-D subscale). *Lactobacillus* counts exhibited a significantly negative correlation with sleep (HAM-D subscale) in the patients with bipolar disorder (ρ = −0.45, *P* = 0.01).

In the patient group, there was no significant partial correlation (adjusted for age and sex) between bacterial counts and HAM-D 17 total score (for *Bifidobacterium*: ρ = −0.06, *P* = 0.72; for *Lactobacillus*: ρ = −0.24, *P* = 0.16) or between bacterial counts and YMRS total score (for *Bifidobacterium*: ρ = 0.11, *P* = 0.53; for *Lactobacillus*: ρ = 0.25, *P* = 0.14). However, subscales of the depressive symptoms according to Seretti et al. (35) (i.e., core, sleep, activity, psychic anxiety, and somatic anxiety) were examined separately (Supplementary Table 1); we found a significantly negative correlation between *Lactobacillus* counts and sleep (ρ = −0.45, *P* = 0.01) (Figure 2) (35).

### Bacterial Counts and Cortisol Levels

**Figure 3:**
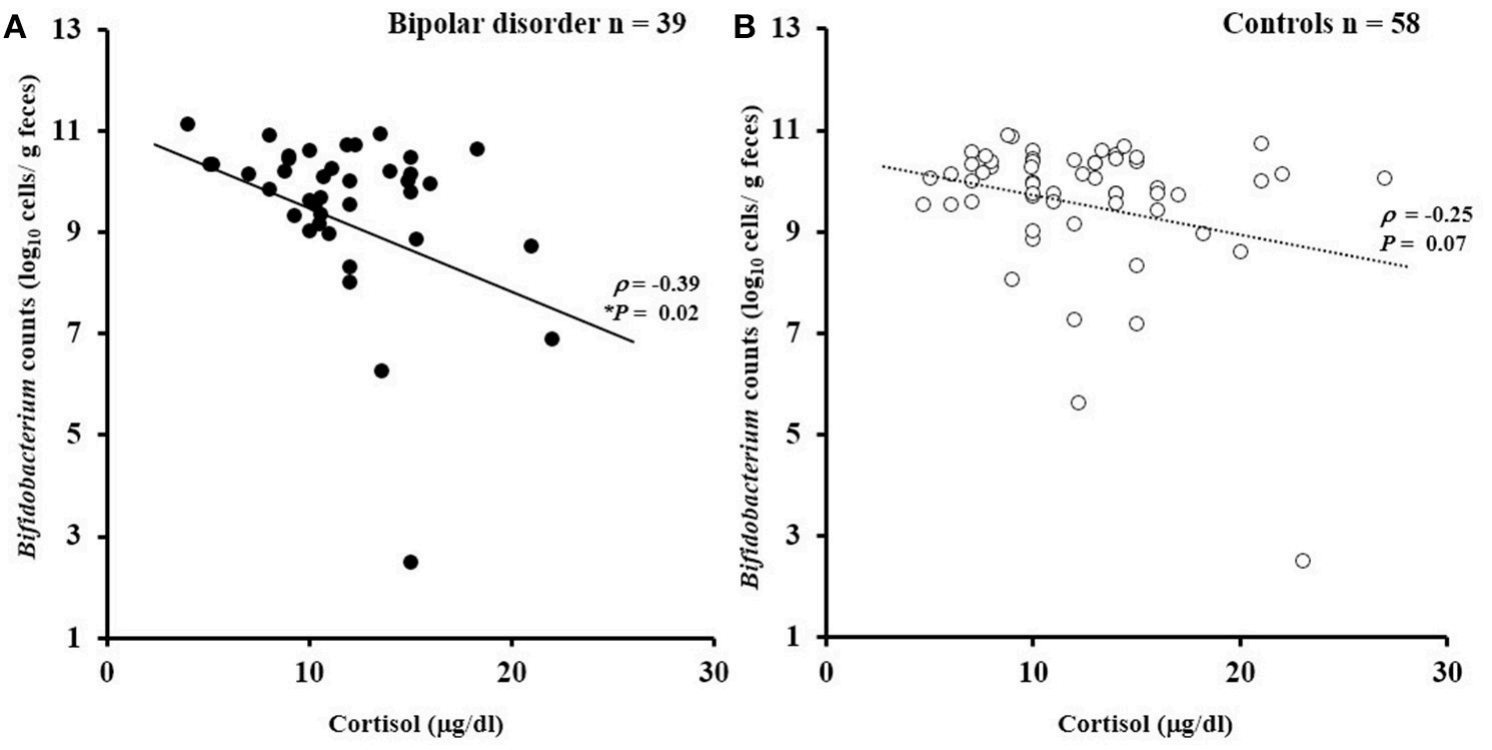
Correlations between *Bifidobacterium* counts and serum cortisol levels. **(A)** *Bifidobacterium* counts exhibited a significantly negative correlation with cortisol levels in the patients with bipolar disorder (ρ = −0.39, *P* = 0.02). **(B)** A similar trend was observed for the controls (ρ = −0.25, *P* = 0.07).

There was no significant difference in serum cortisol levels between the patients and controls (11.8 ± 3.9 μg/dl for the patients vs. 12.7 ± 5.3 μg/dl for the controls; *df* = 1, 90; *F* = 1.75, *P* = 0.19). However, a significant ANCOVA main effect of cortisol levels was observed for *Bifidobacterium* counts (*df* = 1, 90; *F* = 9.4, *P* = 0.003). Figure 3 illustrates the partial correlation between *Bifidobacterium* counts and cortisol levels. A significant negative correlation (age- and sex-adjusted partial correlation) was observed between *Bifidobacterium* counts and cortisol levels in the patients (ρ = −0.39, *P* = 0.02). A similar trend was observed in the controls, although the correlation failed to reach statistical significance (ρ = −0.25, *P* = 0.07). *Lactobacillus* counts exhibited no significant correlation with cortisol levels in the patients (ρ = −0.06, *P* = 0.56) or in controls (ρ = 0.14, *P* = 0.29).

### Bacterial Counts and Medication

There was no significant partial correlation (age- and sex-adjusted) of bacterial counts with dose of antipsychotics (*Bifidobacterium*: ρ = −0.09, *P* = 0.79; *Lactobacillus*: ρ = 0.17, *P* = 0.62) or antidepressants (*Bifidobacterium*: ρ = 0.01, *P* = 0.98; *Lactobacillus*: ρ = 0.07, *P* = 0.86). No significant differences were observed in *Bifidobacterium (df* = 1, 34; *F* = 0.57, *P* = 0.46) or *Lactobacillus* counts (*df* = 1, 34; *F* = 1.08, *P* = 0.31) between users and non-users of any mood stabilizer.

As described above, we included 9 patients who received probiotic agents, which may have minimized the difference in bacterial counts between patients and controls. Even after excluding these 9 patients, however, there was no significant difference for either bacterial count (for *Bifidobacterium: df* = 1, 83; *F* = 0.002, *P* = 0.96, Partial η^2^ = 0.00; for *Lactobacillus: df* = 1, 83; *F* = 0.05, *P* = 0.82, Partial η^2^ = 0.001).

## Discussion

The aim of this study was to first determine whether *Bifidobacterium* and *Lactobacillus* counts in the gut microbiota differ between patients with bipolar disorder and healthy controls. We then examined the correlations between these *Bifidobacterium* and *Lactobacillus* counts and depressive symptoms and serum cortisol levels. We observed no significant difference in *Bifidobacterium* or *Lactobacillus* counts between the two groups. These counts showed no significant correlation with overall severity of manic or depressive symptoms. However, there was a negative correlation between *Lactobacillus* counts and sleep. We found a negative correlation between *Bifidobacterium* counts and cortisol levels in the patients.

To our knowledge, this is the first study that focused on *Bifidobacterium* and *Lactobacillus* counts in the gut microbiota of bipolar patients using the RT-qPCR method. Notably, unlike the current RT-qPCR method, the ordinary sequencing-based (OTU) level analysis cannot precisely obtain data on *Lactobacillus* counts. Since we previously found reduced counts for both bacteria in patients with major depressive disorder compared with controls (21), we expected similar findings in bipolar patients. However, we found no significant difference in bacterial counts between bipolar patients and controls, suggesting that *Bifidobacterium* and *Lactobacillus* counts in the gut may not play a major role in the pathophysiology of bipolar disorder in our sample. Our results are consistent with Evans et al. (23) who reported no significant difference in the ratio of *Bifidobacterium* counts between bipolar patients and controls based on their OTU analysis. Painold et al. (25) reported a significant difference in the phylum *Actinobacteria* to which *Bifidobacterium* belongs; however, they did not report *Bifidobacterium* counts (23, 25). A limitation in our sample is that most patients were being treated with medication and their disease severity was relatively mild (mean HAMD-17 score of 10.3 ± 7.0 and Young mania rating scale score of 2.1 ± 3.5), which may have contributed to the negative results.

We found no significant correlation between overall HAMD-17 or YMRS score and bacterial counts. However, there was a negative correlation between the *Lactobacillus* counts and sleep on the HAM-D scale. In concordance with this, Takada et al. reported that *Lactobacillus casei* had beneficial effects on stress-induced sleep disturbance in humans (36). Miyazaki et al. also found that *Lactobacillus brevis* had beneficial effects on sleep rhythms in mice (37). Together with our finding, increasing *Lactobacillus* counts in the gut may be beneficial to sleep disturbances in bipolar disorder.

Although a recent study reported an association between atypical antipsychotics and specific representation of gut bacterial families in the microbiota, such as *Lachnospiraceae, Akkermansia*, and *Sutterella* in bipolar patients (24), we found no evidence for the effects of any psychotropic medications on *Bifidobacterium* or *Lactobacillus* counts in our subjects.

Interestingly, in the patients, *Bifidobacterium* counts correlated negatively with serum cortisol levels, a hormone critically involved in the HPA axis and commonly regarded as a biological indicator of stress in most psychobiological research (38). A similar non-significant trend (*p* = 0.07) was observed in the controls. Cortisol is involved in the interaction between the brain and various physiological systems, and elevated levels of cortisol have been observed in bipolar disorder (39). As mentioned above, Messaoudi et al. (19) reported that consumption of *Bifidobacterium longum* R0175 and *Lactobacillus helveticus* R0052 for 30 days ameliorated distress symptoms and decreased urinary free cortisol levels in healthy volunteers (19). Allen et al. (40) reported that consumption of *Bifidobacterium longum* 1714 for 4 weeks attenuated stress response to a stressful task in healthy volunteers as reflected by attenuated subjective anxiety and lowered salivary cortisol output (40, 41). Our finding of the negative correlation between *Bifidobacterium* counts and serum cortisol levels is in line with the possible beneficial role of *Bifidobacterium* on stress response. Although the mechanisms underlying the effect of probiotics on cortisol levels remain unclear, animal studies have shown that the administration of probiotics reduced gut permeability, endotoxemia, neuroinflammation, and hypothalamic expression of corticotropin releasing hormone (CRH), which were accompanied by reduced glucocorticoid levels (7, 42). Another study by Takada et al. showed in rats that pretreatment with *Lactobacillus casei* strain, Shirota, suppressed stress-induced increases in plasma corticosterone, and reduced the number of CRH-expressing cells in the periventricular nucleus of the hypothalamus (43). Further, these changes were possibly related with an increase in gastric vagal afferent activity, suggesting the possible involvement of vagus nerve signaling.

### Limitations

Our study has several limitations. First, as mentioned above, the severity of bipolar disorder in the subjects was relatively mild and further studies are necessary to examine whether severe bipolar cases are related to bacterial counts. Second, we compared only the total counts of *Bifidobacterium* and *Lactobacillus*. Both *Bifidobacterium* and *Lactobacillus* are comprised of many species, and health-promoting effects may differ depending on the specific species or strains (44). To elucidate the pathophysiological roles of gut microbiota, further studies on other bacteria counts are warranted. Third, the cross-sectional design of the present study makes it difficult to determine whether the observed relationships were causes or effects of the illness. Finally, although we examined the possible correlation between bacterial counts and current medication doses, we did not consider duration of pharmacological treatment in the analyses.

## Conclusion

We found no significant difference in *Bifidobacterium* or *Lactobacillus* counts between patients with bipolar disorder and controls, suggesting that these bacteria may not play a major role in the pathophysiology of bipolar disorder in our sample. However, the observed negative correlation between *Lactobacillus* counts and sleep and that between *Bifidobacterium* counts and serum cortisol levels point to the possible roles of these bacteria in sleep and stress response in the patients.

## Author Contributions

EA, HT, TA, TaT, and HK conceived and designed the experiments, analyzed the data, and contributed to the writing of the manuscript. ToT, MO, SY, KH, and NK recruited the subjects, assessed psychiatric symptoms, and gave critical comments to the manuscript.

### Conflict of Interest Statement

HT, TA, and TaT are employees of Yakult Honsha Co., Ltd. The remaining authors declare that the research was conducted in the absence of any commercial or financial relationships that could be construed as a potential conflict of interest.

## Abbreviations

HPA: hypothalamic-pituitary-adrenal
NCNP: National Center of Neurology and Psychiatry.
