# Supplementary material for: Bifidobacterium and Lactobacillus Counts in the Gut Microbiota of Patients With Bipolar Disorder and Healthy Controls
Source: Front Psychiatry. 2019 Jan 18;9:730. doi: 10.3389/fpsyt.2018.00730 (PMC6346636; doi:10.3389/fpsyt.2018.00730)
Supplement: Supplementary file 1 [file Table_1.pdf]

Supplemental Table 1. Correlations between bacterial counts and subscales of the depressive symptoms in the patient group.

HAM-D		core	Sleep	Activity	Anxiety	Psychic-anxiety	Somatic-anxiety
Bifidobacterium	***ρ***	-0.07	-0.15	-0.07	0.47	-0.03	0.08
	P	0.68	0.39	0.69	0.79	0.86	0.65
Lactobacillus	***ρ***	-1.0	-0.45	-0.12	-0.09	0.04	-0.14
	P	0.56	0.01*	0.47	0.60	0.80	0.41

Bifidobacterium counts was no significant partial correlation (adjusted for age and sex) between bacterial counts and subscales of the depressive symptoms.

There was significant negative correlation between *Lactobacillus* counts and sleep (HAM-D subscale).
