# Supplementary material for: Bifidobacterium and Lactobacillus Counts in the Gut Microbiota of Patients With Bipolar Disorder and Healthy Controls
Source: Front Psychiatry. 2019 Jan 18;9:730. doi: 10.3389/fpsyt.2018.00730 (PMC6346636; doi:10.3389/fpsyt.2018.00730)
Supplement: Supplementary file 2 [file Image_1.pdf]

Supplementary Figure 1. Bacterial counts for male and female participants in the patient and control groups.

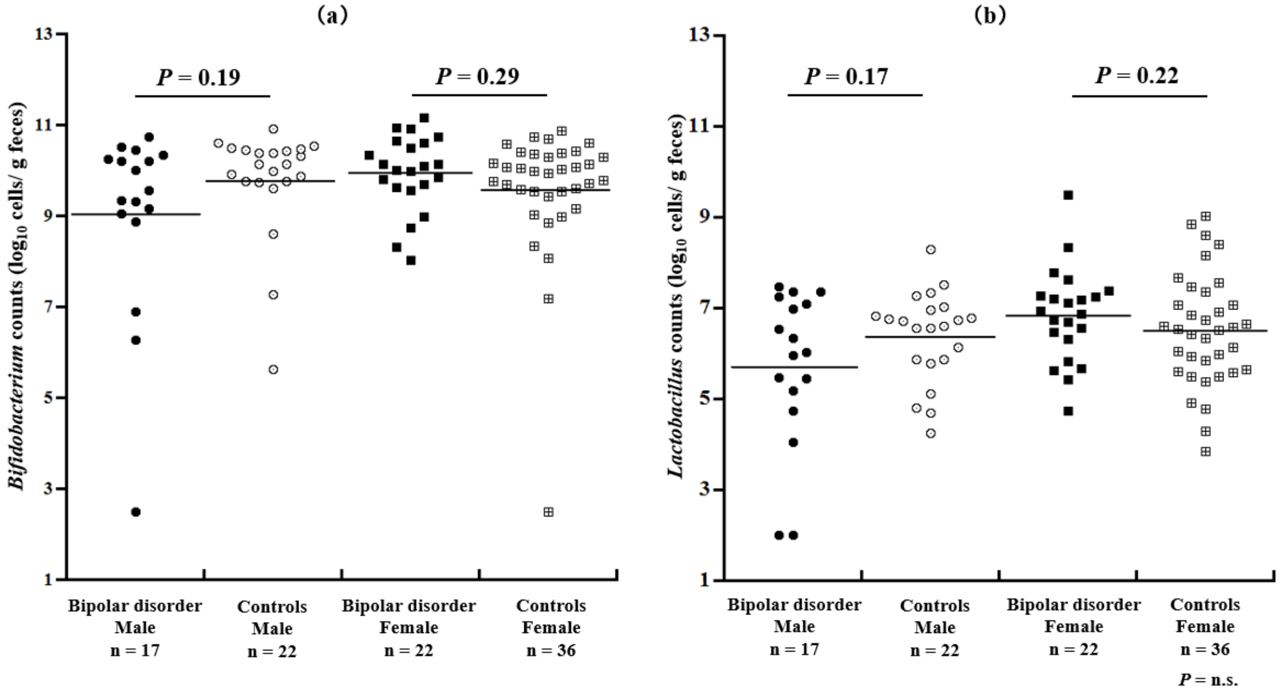

(a): *Bifidobacterium* counts. There was no significant difference in any of the comparisons. Male patients versus male controls ($P = 0.19$); female patients versus female controls ($P = 0.29$).

(b): *Lactobacillus* counts. There was no significant difference between male patients versus male controls ($P = 0.17$), or female patients versus female controls ($P = 0.22$).
